# Radiation Damage in Polyethylene Naphthalate Thin-Film Scintillators

**DOI:** 10.3390/ma15196530

**Published:** 2022-09-21

**Authors:** Marcello Campajola, Francesco Di Capua, Pierluigi Casolaro, Ettore Sarnelli, Alberto Aloisio

**Affiliations:** 1 Istituto Nazionale di Fisica Nucleare (INFN), Sezione di Napoli, Via Cintia 21, I-80126 Naples, Italy; 2 Department of Physics “E. Pancini”, University of Naples “Federico II”, Via Cintia 21, I-80126 Naples, Italy; 3 Albert Einstein Center for Fundamental Physics (AEC), Laboratory for High Energy Physics (LHEP), University of Bern, Sidlerstrasse 5, CH-3012 Bern, Switzerland; 4 CNR-SPIN, Via Campi Flegrei 34, I-80078 Pozzuoli, Italy

**Keywords:** plastic scintillators, radiation damage, polyethylene-naphthalate, thin-film scintillator

## Abstract

This paper describes the scintillation features and the radiation damage in polyethylene naphthalate 100 µm-thick scintillators irradiated with an 11 MeV proton beam and with a 1 MeV electron beam at doses up to 15 and 85 Mrad, respectively. The scintillator emission spectrum, optical transmission, light yield loss, and scintillation pulse decay times were investigated before and after the irradiation. A deep blue emission spectrum peaked at 422 nm, and fast and slow scintillation decay time constants of the order of 1–2 ns and 25–30 nm, respectively, were measured. After irradiation, transmittance showed a loss of transparency for wavelengths between 380 and 420 nm, and a light yield reduction of ~40% was measured at the maximum dose of 85 Mrad.

## 1. Introduction

Plastic scintillators are well-established radiation detectors in several applications, including particle and nuclear physics experiments, as well as medical physics. They feature a fast decay time, high light yield (LY) and optical transmission, ease of manufacture and low cost [1,2,3]. Plastic scintillators are typically made of a polymer matrix with small quantities of primary and secondary dyes [4]. When the polymer base molecules are excited by the radiation, the excitation energy is promptly transferred to the primary dye via the Foerster mechanism [5], and thus to the secondary dye (also referred to as wavelength shifter) that emits photons in a wavelength range detectable from most of the typically used photodetectors. Typical plastic scintillators’ polymer bases are aromatic hydrocarbon chemical compounds such as polystyrene (PS) or polyvinyltoluens (PVT). These are doped with fluorescent molecules such as 2,5-Diphenyloxazole (PPO) and 1,4-bis (5-phenyloxazol-2-yl) benzene (POPOP), typically in percentages of ~1% and ~0.005%, respectively [6,7].

**Figure 1:**
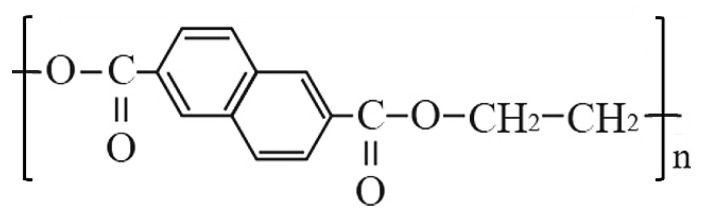
Chemical formula of the polyethylene naphthalate.

Recent works proved that undoped aromatic ring polymers commonly known as low-cost plastic materials, such as polyethylene naphthalate (PEN) or polyethylene terephthalate (PET), are good scintillation materials [8,9,10,11]. PEN is synthesized by the poly-condensation of dimethyl-2,6-naphthalenedicarboxylate and ethylene glycol. Its monomeric chemical structure consists of a double benzene ring, as shown in Figure 1.

PEN features blue light emission with a peak at ~420 nm and a high LY of ~10^4^ photons/MeV [9], a short decay time [12,13], and a moderately high density (1.33 g/cm^3^) with respect to other common plastic scintillators, despite a relatively high refractive index [14]. Moreover, PEN has good mechanical properties that allow its production in different sizes and shapes, including thin flexible films, which could be useful in applications where curved surfaces must be instrumented. Because of these properties and ease of manufacture, PEN has recently drawn the attention of the scientific community for its use as a scintillator. As an example, some works explored its suitability for dosimetry in nuclear and medical physics [15,16,17] and calorimetry in collider experiments [18]. PEN is also suitable for neutron detection due to the high amount of hydrogen, which scatters neutrons through elastic scattering on protons [19]. PEN has also been proposed as a wavelength shifter for noble liquid scintillators in astroparticle underground experiments, as it fulfills the requirements for high purity and low background contamination [20,21,22].

Some of these applications require the detectors to operate in environments with high radiation levels while asking for long-term stability. Specifically, accelerator or collider applications demand resistance to extremely high radiation doses. As an example, the radiation background in the hadronic calorimeter of the CMS detector at the Large Hadron Collider (LHC) results in a 10 Mrad expected dose after 10 years of operation [23]. Ongoing upgrades of existing high-energy physics experiments and future particle colliders will deal with even higher radiation dose levels [24]. During operation in such harsh environments, the interaction of the radiation with the scintillation detectors may result in the change of their physical and chemical properties through several mechanisms depending on the selected material. Possible effects include polymer chain scission, breakage of bonds, cross-linking and the formation of new chemical bonds [23]. These can affect either the scintillation light mechanisms or the light transmission, resulting in a deterioration of the detector performances [25,26,27,28,29,30,31]. Therefore, the search for new scintillation materials capable of withstanding high radiation doses is of paramount importance.

In this work, we describe the scintillation features and the radiation damage in a 100 µm-thick polyethylene naphthalate scintillator. We irradiated several samples of such a scintillator with an 11 MeV proton beam and with a 1 MeV electron beam at the maximum doses of 15 Mrad and 85 Mrad, respectively. We characterized the PEN emission spectrum, optical transmission, scintillation decay time and LY loss before and after the irradiation.

Our study adds to the few other works on radiation damage in PEN [32,33,34,35], and explores radiation effects on several PEN properties over a broader range of doses, with two radiation types (electrons and protons) at two different energies, paving the way for using such thin-film scintillators in applications where high levels of radiation are expected.

## 2. Materials and Methods

**Figure 2:**
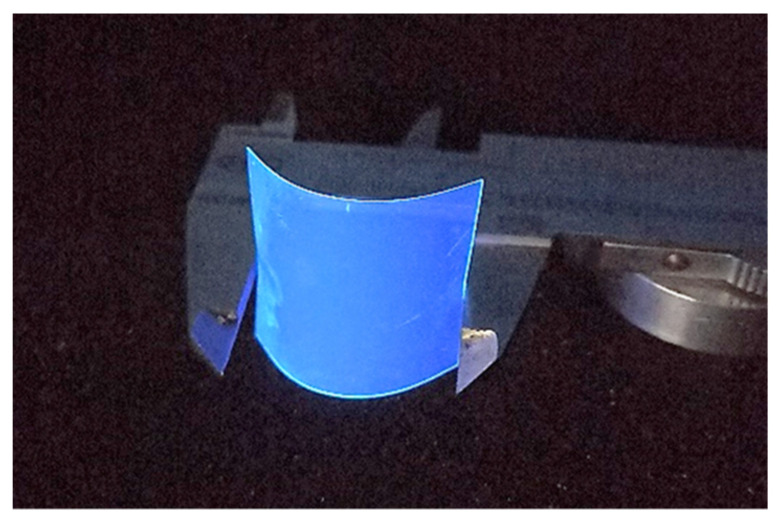
A 30 × 40 × 0.1 mm^3^ thin-film flexible PEN scintillator illuminated with UV light.

The PEN scintillator under investigation is produced by Teonex^®^ as a 100 µm-thick flexible film (mod. Q65HA). Starting from the commercialized format, we obtained, by cutting, samples of 30 × 40 mm^2^, as shown in Figure 2.

Two irradiation campaigns were performed, one with a proton beam and the other with an electron beam. In both cases, PEN samples were irradiated in air (at room temperature), placed at the exit of the beam-line and exposed frontally to the beam. The proton irradiation was carried out at the Tandem accelerator of the INFN Laboratori Nazionali del Sud. Considering the energy loss in the beam extraction window and in the air layer between the end of the beam line and the sample, the proton energy on the sample surface was estimated to be 11 MeV. The PEN samples were exposed to a maximum proton dose of 15 Mrad (to water), with a max dose rate of 21 krad/min. The electron irradiation was performed at the ILU-6 accelerator at the Institute of Nuclear Chemistry and Technology (INCT) in Warsaw (Poland) with a 1 MeV electron beam. The samples were exposed to a maximum dose of 85 Mrad (to water) with a max dose rate of 5 Mrad/min. In both irradiations, the beams had enough energy to pass through the whole scintillator: the expected penetration ranges in polyethylene at those energies are ~1.1 mm and ~3.5 mm for protons and electrons, respectively.

The emission spectrum was measured with a spectrometer (mod. AvaSpac-2048XL from Avantes, Apeldoorn, The Netherlands) by stimulating PEN emission with a 280 nm LED source operating at 20 mW. The optical transmittance in the 300–800 nm wavelength range was measured with the same spectrometer by using a deuterium/halogen light source.

**Figure 3:**
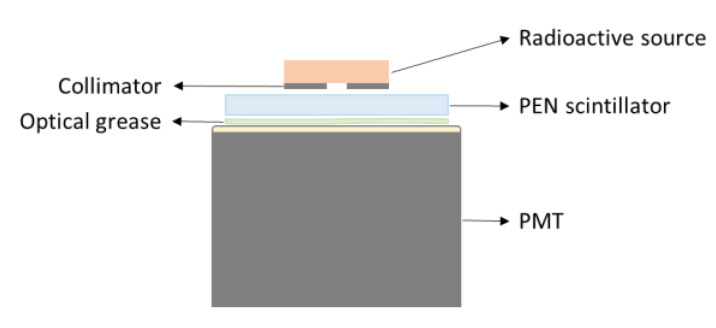
Schematic representation of the experimental setup for the PEN thin-film scintillators light yield measurement.

To study the scintillation pulse decay times and the LY, we exposed our PEN samples to both ^241^Am and ^137^Cs sources. The former decays to the second excited state of ^237^Np with 85% absolute intensity by emitting 5.486 MeV alpha particles; the latter decays to ^m137^Ba with 95% absolute intensity by emitting electrons with maximum energy of 0.514 MeV. Scintillation pulses were measured by coupling the PEN scintillator to a flat window Hamamatsu R5900 PhotoMultiplier Tube (PMT) with a gain of ~$4\times 10^{6}$ biased at 900 V. The radioactive source was positioned on top of a collimator with a 2 mm hole diameter. A schematic of the experimental setup is shown in Figure 3.

The scintillation pulse decay times were measured by acquiring the signal waveform directly from the PMT with a 1 GHz-bandwidth, 10 GS/s sampling rate LeCroy oscilloscope. As for the measurement of the signal spectra and of the LY, PMT signals were amplified and acquired via an ORTEC EASY-MCA Multichannel Analyzer (MCA).

## 3. Results

### 3.1. Preliminary Characterization

**Figure 4:**
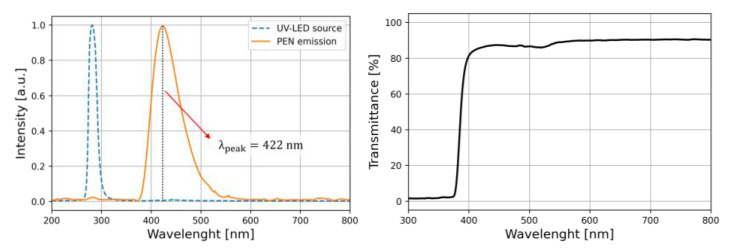
(**Left**): Emission spectrum of the PEN scintillator (orange) stimulated by a UV LED source (blue dashed curve). (**Right**): PEN transmittance relative to air as a function of the wavelength.

We tested the scintillation features of our 100 µm-thick film PEN samples prior to the irradiation. The samples were stimulated with a 285 nm LED and the emission spectrum was measured, as shown in Figure 4 (left). A single peak feature at 422 nm was observed, in agreement with the values reported in the literature [9]. Such a deep blue emission spectrum matches the high-quantum-efficiency spectral regions of many commercially available photomultipliers or SiPMs [36,37]. The optical transmittance, relative to air, was measured in the range from 300 to 800 nm, as shown in Figure 4 (Right). Transmittance exhibits a steep decrease for wavelengths lower than about 400 nm and is negligible below 370 nm.

**Figure 5:**
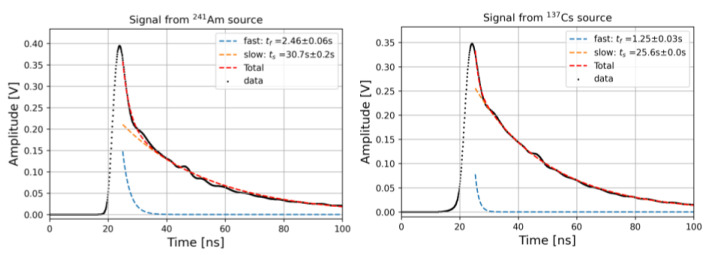
Signal waveform of PEN scintillators excited with ^241^Am (**left**) and ^137^Cs (**right**) sources. The signals shown are the averages of 10,000 waveforms.

Scintillation decay time constants were measured by exciting the scintillator with alpha and beta particles from ^241^Am and ^137^Cs sources and coupling it to the PMT according to the description given in Section 2. The scintillation light pulses over 10,000 waveforms averages are shown in Figure 5.

The signal decay shape was modeled as the sum of two negative exponential functions with time constants $\tau_{fast}$ and $\tau_{slow}$, respectively, which account for different de-excitation mechanisms [3]:

(1) $$\mathrm{Amplitude}\;\left(t\right)=\frac{A}{\tau_{fast}}e^{-\frac{t}{\tau_{fast}}}+\frac{B}{\tau_{slow}}e^{-\frac{t}{\tau_{slow}}}$$

By fitting the experimental data for a pristine PEN sample with Equation (1), we observed fast and slow time constants of 2.46 ns and 30.7 ns for the sample excited with ^241^Am and of 1.25 ns and 25.6 ns for the sample excited with ^137^Cs. Similar values have been reported in the literature for the slow decay time constants [12,13], while the fast component had not been measured before. Such small time constants strongly increase the interest in using such materials in many applications requiring good timing resolution.

### 3.2. Post-Irradiation Measurements

After the irradiation, PEN samples were kept in the dark with a controlled environment temperature of 25 °C. All post-irradiation measurements were performed one month after the irradiation campaigns in order to measure the persistent damage effects only. Indeed, a partial recovery mechanism of the radiation-induced damage in PEN with a characteristic time of ~10 days is reported in the literature [32].

Differences in the emission spectrum before and after the irradiation are below 0.5%. This is in agreement with the results obtained with gamma-irradiated PEN scintillators reported in Ref. [33], where only a shift of a few nanometers of the emission peak was observed for a dose of 65 Mrad.

Similarly, no degradation trend in the decay times was observed as a function of the dose, with variations within 7%. This suggests that the damage to the light emission mechanisms is negligible up to the considered doses.

**Figure 6:**
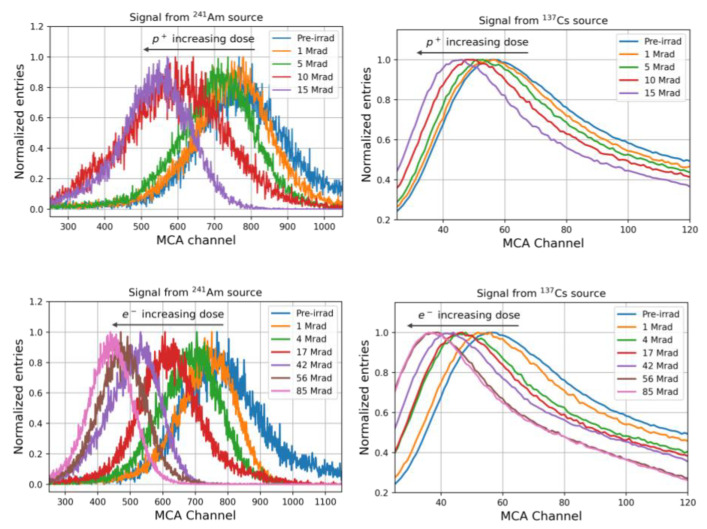
Light yield spectra of PEN scintillators irradiated with protons (**top**) and electrons (**bottom**), measured upon excitation with ^241^Am (**left**) and ^137^Cs (**right**) sources.

The light yield spectra of the irradiated samples are shown in Figure 6. The left plots show the spectra measured by using the ^241^Am source. A typical distribution of a monochromatic particle losing all the energy in the detector was observed; indeed, 5.486 MeV alpha particles from ^241^Am have a range of ~35 µm in PEN. In the right plots, spectra measured using ^137^Cs source are shown. The observed distribution is due to the energy loss of beta particles with a continuum energy spectrum, which accounts for the observed long tail.

The effect of the irradiation is observable as a decrease in the peak centroids as the radiation dose increases.

**Figure 7:**
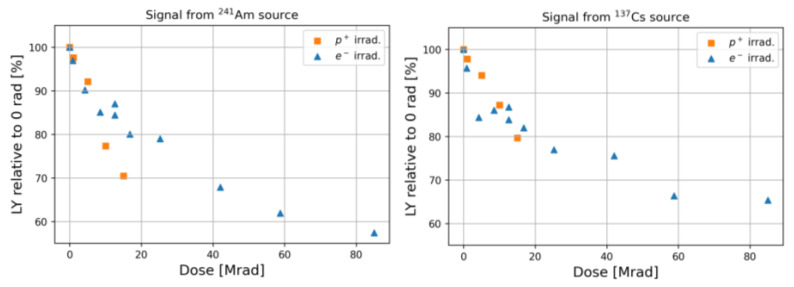
Irradiated PEN light yield normalized to that of a pristine scintillator as a function of the delivered dose for proton and electron irradiations. Scintillation light was measured upon excitation with (**left**) ^241^Am and (**right**) ^137^Cs sources.

In Figure 7, the irradiated samples LY normalized to that of a pristine scintillator as a function of the delivered dose are shown. Similar trends for both proton and electron irradiated samples can be observed as long as the same dose range is investigated. A slightly larger LY degradation is observed when measured with ^241^Am than with ^137^Cs. This could be ascribed to the fact that alpha particle-induced scintillation occurs near the surface of PEN samples, and thus the scintillation light is more attenuated than that due to ^137^Cs beta particles, which excite the sample along with its entire thickness. At higher doses, the radiation damage rate slows down and LY loss reaches values close to 40%.

In Ref. [32], the authors find, for a 1 mm-thick PEN scintillator irradiated with gamma at a dose of 14 Mrad, a permanent ~20% LY loss, which is close to our findings at similar doses. A larger degradation is observed in Ref. [34], where after irradiation of a 9 µm-thick PEN film with 1 MeV protons, a ~55% loss was measured at a dose of ~150 Mrad after 3 h from the irradiation.

**Figure 8:**
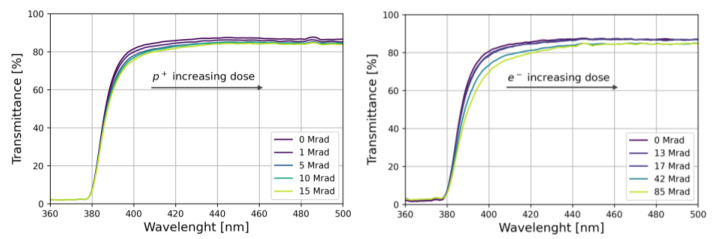
Light transmission spectra for PEN scintillators irradiated with protons (**left**) and electrons (**right**).

Optical transmission measurements were repeated after irradiation as a function of the delivered dose. The results are shown in Figure 8. As the dose increases, the absorption edge shifts to longer wavelengths and an overall decrease in the transmittance is observed.

**Figure 9:**
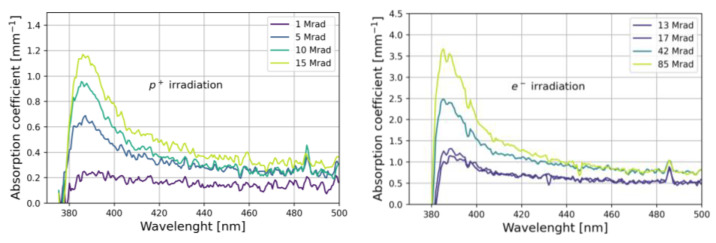
Radiation-induced absorption coefficient spectra for samples irradiated with protons (**left**) and electrons (**right**).

We computed the radiation-induced absorption coefficient μ as a function of the wavelength as:

(2) $$\mu =\ln\left(\frac{T_{b}}{T_{a}}\right)/d,$$

where $T_{b}$ and $T_{a}$ are the transmission measured before and after irradiation, respectively, and *d* is the sample thickness (*d* = 100 μm). The results are shown in Figure 9, where large absorption values are observed in the 380–420 nm-wavelength region, suggesting that the effect of irradiation is the production of free radicals acting as absorption centers [38].

## 4. Conclusions

In recent years, PEN has emerged as a good scintillation material due to its behaviors such as high light yield and blue emission, which match well with the sensitivity range of many common photodetectors. Furthermore, PEN is a common and cheap material and can be produced in different sizes and shapes, including in the form of flexible films.

In this work, we report on the characterization of the scintillation features and the radiation damage in polyethylene naphthalate 100 µm-thick scintillators.

Our results revealed the excellent PEN scintillation features: a deep blue emission spectrum peaked at 422 nm and small fast and slow decay time constants of the order of 1–2 ns and 25–30 nm, respectively.

The radiation damage in PEN has been investigated by performing two irradiation tests with 11 MeV protons and 1 MeV electrons with maximum doses of 15 Mrad and 85 Mrad, respectively. Irradiation did not change the light emission spectrum and nor the scintillation decay time constants. A reduction of ~40% in the light yield was observed at the maximum delivered dose of 85 Mrad. Transmittance measurements showed a marked loss of transparency for wavelengths between 380 and 420 nm.

Based on the findings reported in this work, we foresee the possibility of using such PEN thin-film scintillators in radiation detection applications where peculiar geometries and high doses are expected.

Future works will be devoted to detailed studies of the PEN damage for different high-dose radiation types, which can be of paramount importance for applications involving the presence of more than one radiation type.

## Data Availability

The data presented in this study are available upon request from the corresponding author.

## References

[1] Leo W.R. (1992). Techniques for Nuclear and Particle Physics Experiments: A How-to Approach.

[2] Knoll G. (2010). Radiation Detection and Measurement.

[3] Buvat I., Grupen C. (2012). Handbook of Particle Detection and Imaging.

[4] Hamel M. (2021). Plastic Scintillators: Chemistry and Applications.

[5] Berlman I.B. (1973). Energy Transfer Parameters of Aromatic Compounds.

[6] Moser S.W., Harder W.F., Hurlbut C.R., Kusner M.R. (1993). Principles and practice of plastic scintillator design. Radiat. Phys. Chem..

[7] Nakamura H. (2012). Development of polystyrene-based scintillation materials and its mechanisms. Appl. Phys. Lett..

[8] Nakamura H., Kitamura H., Hazama R. (2010). Radiation measurements with heat-proof polyethylene terephthalate bottles. Proc. R. Soc. A.

[9] Nakamura H., Shirakawa Y., Takahashi S., Shimizu H. (2011). Evidence of deep-blue photon emission at high efficiency by common plastic. Europhys. Lett..

[10] Nakamura H., Shirakawa Y., Kitamura H., Yamada T., Shidara Z., Yokozuka T., Nguyen P., Takahashi T., Takahashi S. (2013). Blended polyethylene terephthalate and polyethylene naphthalate polymers for scintillation base substrates. Radiat. Meas..

[11] Nakamura H., Yamada T., Shirakawa Y., Kitamura H., Shidara Z., Yokozuka T., Nguyen P., Kanayama M., Takahashi S. (2013). Optimized mounting of a polyethylene naphthalate scintillation material in a radiation detector. Appl. Radiat. Isot..

[12] Wetzel J.W., Tiras E., Bilki B., Koseyan O., Bostan N., Onel Y. Measuring the Scintillation Decay Constant of PEN and PET with 120 GeV Proton Beam Excitation. Proceedings of the 2020 IEEE Nuclear Science Symposium and Medical Imaging Conference (NSS/MIC).

[13] Wetzel J., Emrah T., Burak B., Nilay B. (2020). Scintillation timing characteristics of common plastics for radiation detection excited with 120 GeV protons. Turkish Jour. Phys..

[14] Nakamura H., Shirakawa Y., Kitamura H., Sato N., Takahashi S. (2014). Detection of alpha particles with undoped poly (ethylene naphthalate). Nucl. Instrum. Methods Phys. Res. A.

[15] Shirakawa Y., Nakamura H., Kamata T., Watai K., Mitsunaga M., Shidara Z., Murakawa F. (2013). Radiation counting characteristics on surface-modified polyethylene naphthalate scintillators. Radioisotopes.

[16] Flühs D., Flühs A., Ebenau M., Eichmann M. (2015). Polyethylene Naphthalate Scintillator: A Novel Detector for the Dosimetry of Radioactive Ophthalmic Applicators. Ocul. Oncol. Pathol..

[17] Garankin J., Plukis A., Plukienė R., Lagzdina E., Remeikis V. (2018). Identification of Particles of Ionizing Radiation by the Analysis of Fluorescence Pulse Form of the Thin Pen Film Scintillator. IEEE Trans. Nucl. Sci..

[18] Tiras E. (2015). Radiation Hard & High Light Yield Scintillator Search for CMS Phase II Upgrade. arXiv.

[19] Garankin J., Plukis A., Lagzdina E. (2017). Thermal neutron detection using thin PEN film doped with high cross section materials. Energetika.

[20] Kuźniak M., Broerman B., Pollmann T., Araujo G.R. (2019). Polyethylene naphthalate film as a wavelength shifter in liquid argon detectors. Eur. Phys. J. C.

[21] Obara S., Gando Y., Ishidoshihro K. (2020). Scintillation balloon for liquid scintillator base Neutrinoless double beta decay search experiments. J. Phys. Conf. Ser..

[22] Efremenko Y., Fajt L., Febbraro M., Fischer F., Hayward C., Hodák R., Kraetzschmar T., Majorovits B., Muenstermann D., Öz E. (2019). Use of poly(ethylene naphthalate) as a self-vetoing structural material. J. Instrum..

[23] Kharzheev Y.N. (2019). Radiation Hardness of Scintillation Detectors Based on Organic Plastic Scintillators and Optical Fibers. Phys. Part. Nuclei.

[24] Apollinari G., Brüning O., Nakamoto T., Rossi L. (2017). High luminosity large hadron collider HL-LHC. arXiv.

[25] Hamada M., Rela P.R., Da Costa F.E., De Mesquita C.H. (1999). Radiation damage studies on the optical and mechanical properties of plastic scintillators. Nucl. Instrum. Methods Phys. Res. Sect. A.

[26] Jivan H., Mdhluli J.E., Sideras-Haddad E., Mellado B., Erasmus R., Madhuku M. (2017). Radiation damage effects on the optical properties of plastic scintillators. Nucl. Instrum. Methods Phys. Res. Sect. B.

[27] Mokgatitswane G., Baranov V., Davydov Y., Erasmus R.M. (2018). Effects of neutron radiation on the optical and structural properties of blue and green emitting plastic scintillators. Nucl. Instrum. Methods Phys. Res. Sect. B.

[28] Li Z., Wu C., Heng Y., Zhao X., Shi F., Sun Z., Wu J., An Z., Zhao Y., Zhang Z. (2005). Properties of plastic scintillators after irradiation. Nucl. Instrum. Methods Phys. Res. Sect. A.

[29] Khachatryan V., Sirunyan A.M., Tumasyan A., Litomin A., Mossolov V., Shumeiko N., Van De Klundert M., Van Haevermaet H., Van Mechelen P., Van Spilbeeck A. (2016). Dose rate effects in the radiation damage of the plastic scintillators of the CMS hadron endcap calorimeter. J. Instrum..

[30] Vasil’chenko V.G., Lapshin V.G., Peresypkin A.I., Konstantinchenko A.A., Pyshchev A.I., Shershukov V.M., Semenov B.V., Solov’ev A.S. (1996). New results on radiation damage studies of plastic scintillators. Nucl. Instrum. Methods Phys. Res. Sect. A.

[31] Jivan H., Sideras-Haddad E., Erasmus R., Liao S., Madhuku M., Peters G., Sekonya K., Solvyanov O. (2015). Radiation hardness of plastic scintillators for the Tile Calorimeter of the ATLAS detector. J. Physics.Conf. Ser..

[32] Wetzel J., Tiras E., Bilki B., Onel Y., Winn D. (2016). Radiation damage and recovery properties of common plastics PEN (Polyethylene Naphthalate) and PET (Polyethylene Terephthalate) using a ^137^Cs gamma ray source up to 1.4 Mrad and 14 Mrad. J. Instrum..

[33] Belkahla N., Teyssedre G., Saidi-Amroun N., Saidi M., Boudou L., Berquez L. (2014). Space charge, conduction and photoluminescence measurements in gamma irradiated poly (ethylene-2,6-naphthalate). Radiat. Phys. Chem..

[34] Nagata S., Mitsuzuka M., Onodera S., Yaegashi T., Hoshi K., Zhao M., Shikama T. (2013). Damage and recovery processes for the luminescence of irradiated PEN films. Nucl. Instrum. Methods Phys. Res. Sect. B.

[35] Campajola M., Di Capua F., Sarnelli E., Aloisio A. (2021). Characterization of the Radiation-Induced Damage in a PEN (Polyethylene Naphthalate) Scintillation Detector. Eng. Proc..

[36] Gundacker S., Heering A. (2020). The silicon photomultiplier: Fundamentals and applications of a modern solid-state photon detector. Phys. Med. Biol..

[37] Piemonte C., Alberto G. (2018). Overview on the main parameters and technology of modern Silicon Photomultipliers. Nucl. Instrum. Methods Phys. Res. Sect. A.

[38] Zhu R. (1998). Radiation damage in scintillating crystals. Nucl. Instrum. Methods Phys. Res. Sect. A.

